# Role of Cone Beam Computed Tomography in Rehabilitation of a Traumatised Deficient Maxillary Alveolar Ridge Using Symphyseal Block Graft Placement

**DOI:** 10.1155/2013/748405

**Published:** 2013-05-22

**Authors:** Shipra Arora, Arundeep Kaur Lamba, Farrukh Faraz, Shruti Tandon, Abdul Ahad

**Affiliations:** Department of Periodontics and Oral Implantology, Maulana Azad Institute of Dental Sciences, New Delhi 110002, India

## Abstract

Deficiencies in the alveolar ridges cause multiple problems in achieving aesthetic and functional outcome of implant therapy and are commonly restored by using onlay graft from intraoral source. Careful assessment of the recipient as well as the donor site using cone beam computed tomography (CBCT) is a prerequisite to ideal treatment planning. This paper highlights the critical role of CBCT in planning a successful rehabilitation of traumatised deficient anterior maxillary alveolar ridge using autogenous block graft from mandibular symphysis, followed by implant placement. A 21-year-old male reported with missing right maxillary lateral incisor due to traumatic avulsion 6 months back. A concavity was found on the labial aspect of edentulous area. Serial transplanar images on CBCT revealed gross irregular radiolucency in place of labial cortical plate. Using CBCT, size of the required block was estimated, and mandibular symphyseal area was evaluated for the feasibility of harvesting a graft of suitable dimension. Onlay block graft was harvested from mandibular symphysis and placed at the edentulous site to augment the alveolar ridge. Implants were placed 5 months later and loaded successfully after osseointegration. After 1 year of followup, implant-based prosthesis is working well, without any complications.

## 1. Introduction

Edentulous sites with insufficient bone volume present a challenge to many clinicians planning an implant based prosthetic rehabilitation. A successful implant therapy with restoration of aesthetics and functions requires sufficient alveolar bone, with acceptable maxillary-mandibular relationship.

In cases of tooth loss due to dentoalveolar fracture, generally, the quantity and quality of the existing ridge are grossly compromised. Thickness of the facial bone wall may directly influence the convexity of the tissues at the emerging implant crown. In anterior maxilla, detailed assessment of the existing bone is required to ensure the satisfactory outcome in terms of aesthetics as well as function. Intraoral periapical (IOPA) and panoramic radiographs often fail to reveal the true morphology of the ridge, particularly a defect in labial cortical plate. Cone beam computed tomography (CBCT) is now a preferred technique for 3-dimensional assessment of alveolar bone anatomy [1]. It offers axial, reformatted panoramic and serial transplanar images at the desired location. CBCT of the jaws helps in detecting the bone morphology at the edentulous site and appropriately planning the augmentation procedure, size of the block required, and feasibility of the donor site at symphysis of the mandible. The technique offers high image resolution of 0.123 mm in addition to improved image quality and much lower radiation exposure compared to conventional computed tomography (CT) [1].

The ideal solution for the problem of deficient alveolar ridge lies in the careful assessment and planning reestablishment of the lost bone volume for a favourable anatomy with suitable lamellar bone for implant stability and aesthetics.

Bone grafting materials and techniques that have been developed to cope with this challenge show promising outcomes. An ideal bone grafting material should be biocompatible, have no rejection response, pose no risk of disease transmission, provide a scaffold promoting bone regeneration, and afford initial mechanical stability maintained throughout the entire treatment time. 

Autogenous bone grafting remains the most predictable and best documented method till date and is considered the gold standard for bone augmentation [2, 3]. The purported osteogenic potential of autogenous block grafts is one of the major considerations in promoting it as the gold standard. However, osteogenic potential may vary among patients, and it decreases with increasing age. The successful healing of block grafts, as with all grafts, depends primarily on the establishment of graft vascularisation. Osteogenesis cannot occur until the graft is vascularised [2].

Gains of 4-5 mm in width and height of alveolar ridge have been reported with autogenous onlay grafts with associated implant success rates [2, 4]. Although the various case reports document the successful placement of implants following ridge augmentation with onlay allografts, the effectiveness and predictability of these procedures for ridge augmentation remain unclear. 

This paper reports a case of CBCT assisted diagnosis and treatment planning of severely deficient maxillary anterior edentulous site and successful augmentation using an onlay block graft from mandibular symphysis followed by prosthetic rehabilitation after implant placement.

## 2. Case Presentation

**Figure 1:**
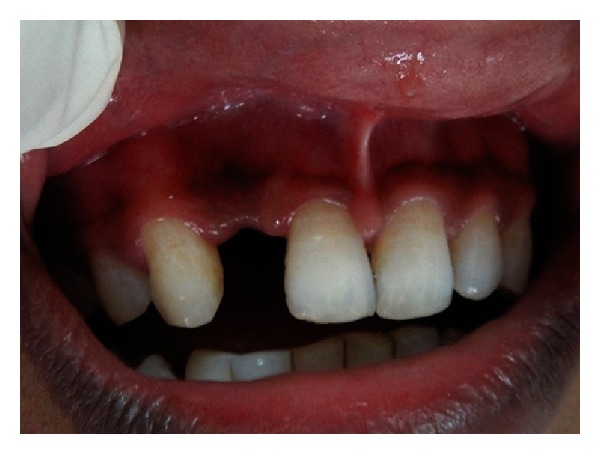
Preoperative view of the edentulous area showing concavity on the labial surface.

**Figure 2:**
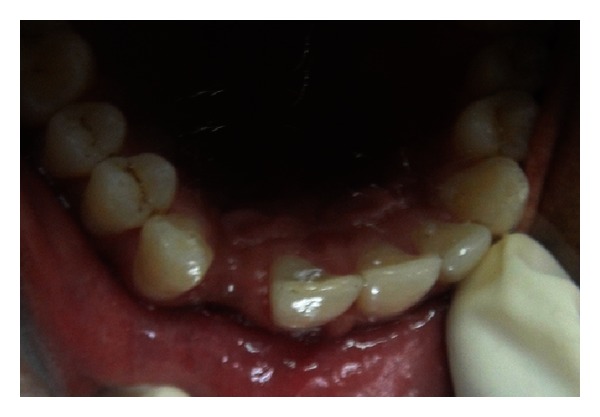
Preoperative occlusal view of the edentulous area.

**Figure 3:**
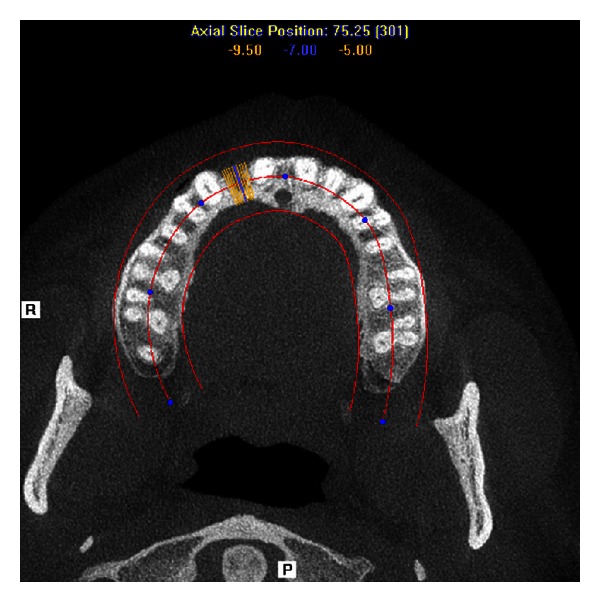
Preoperative axial section on CBCT. Decreased labio-palatal thickness of edentulous area may be noted.

**Figure 4:**
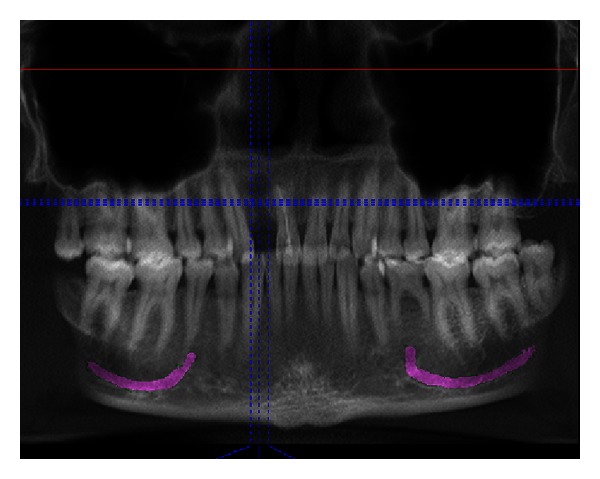
Preoperative CBCT with reformatted panoramic view. Gross radiolucency may be noted at the edentulous site.

**Figure 5:**
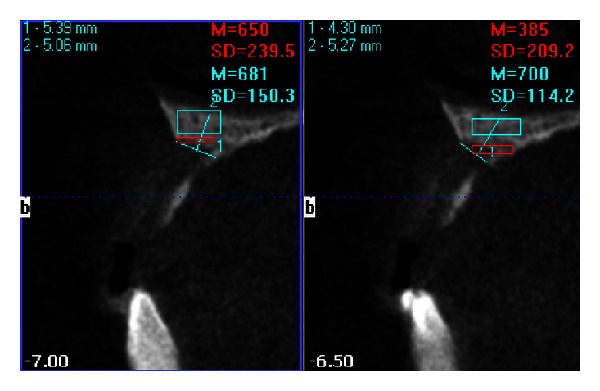
Transplanar images of of edentulous area on CBCT. Gross deficiency in dimensions of alveolar ridge is evident.

A 21-year-old male reported to the Maulana Azad Institute of Dental Sciences, New Delhi, India, with the chief complaint of missing upper front tooth for the last 6 months. Past history revealed that the tooth got avulsed due to contact sports 6 months back. However, patient reported no history of any fracture of the jaw bone. He was a nonsmoker, and there was no significant history of any chronic systemic disease. Intraoral examination showed that his oral hygiene was good. As per his chief complaint, tooth number 12 was missing. The tooth number 11 had a restoration on palatal surface. Due to the trauma, substantial hard and soft tissue loss had occurred, and an “hour glass” concavity could be noted on the labial surface of edentulous site (Figure 1). On ridge mapping the width of the bone at the centre of concavity was found to be only 3.7 mm which was insufficient for implant placement (Figure 2). To get a precise view, a CBCT of maxilla and mandible was obtained (Figures 3 and 4). On cross-sectional slice through middle of edentulous area, using a software (iCATVision—Imaging Sciences International), gross radiolucency was observed in place of labial cortical plate (Figure 5). Amount of alveolar bone at the edentulous site was clearly insufficient for successful placement of an implant of appropriate dimension.

Transplanar images of mandibular symphysis were evaluated to assess the feasibility of harvesting the required block. Labiolingual thickness, length of roots of mandibular incisors, and position of incisive nerve were found to be favourable.

### 2.1. Management of the Case

A surgical procedure was planned under local anaesthesia, after obtaining a written informed consent from the patient. Since the labiopalatal dimension was insufficient for correct placement of implant, onlay block grafting was planned. Mandibular symphysis was chosen as the donor site.

#### 2.1.1. Preparation of the Recipient Site

**Figure 6:**
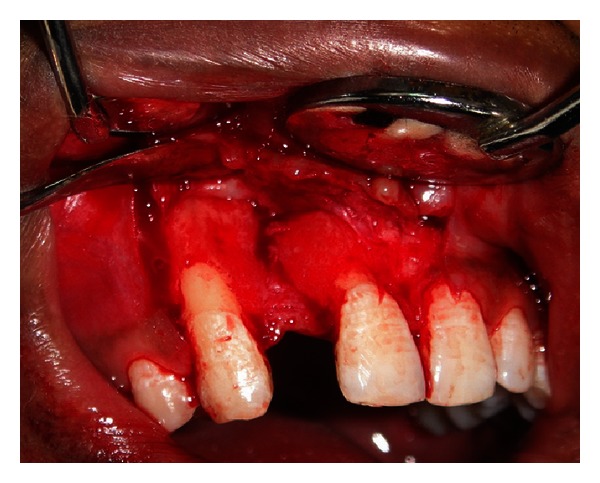
Defect at the edentulous site as viewed on surgical exposure.

After administering infraorbital nerve block using Lignocaine 2% (with Adrenaline 1 : 80,000), midcrestal incision on the edentulous area and two releasing incisions were given to reflect the mucoperiosteal flap. On reflection of flap, concavity was observed on the labial aspect of edentulous ridge (Figure 6). Width of the ridge was found to be 3.6 mm, 3 mm apical to the alveolar crest.

#### 2.1.2. Harvesting of Block Graft from Mandibular Symphysis

**Figure 7:**
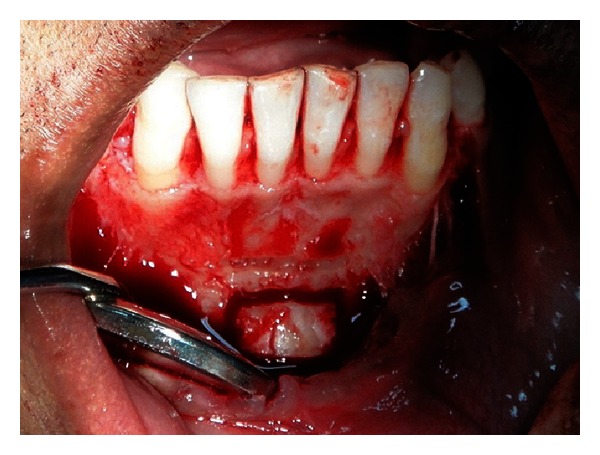
Outline of the block graft harvested from mandibular symphysis.

Bilateral mental nerve blocks (Lignocaine 2% with Adrenaline 1 : 80,000) were given, followed by sulcular incision on labial aspect of mandibular anterior teeth, extending from mesial of right first premolar to the left one. Two releasing incisions were given to reflect the mucoperiosteal flap. Flap was extended up to 2 cm apically from the alveolar crest. A 5 mm × 7 mm outline was marked at 12 mm apical to alveolar crest to avoid iatrogenic trauma to root apices of mandibular incisors (Figure 7). A piezo surgical unit (Italia Medica, Milano, Italy) was then used to harvest the bone block with a thickness of 4 mm. Osteotomy site was filled with Decalcified Freeze Dried Bone Allograft (DFDBA) of particle size 500–1000 *μ*m (Tata Memorial Hospital Tissue Bank, Mumbai), and flap was sutured with 3-0 silk (Ethicon, Johnson & Johnson Ltd.)

#### 2.1.3. Placement of Block Graft at the Recipient Site

**Figure 8:**
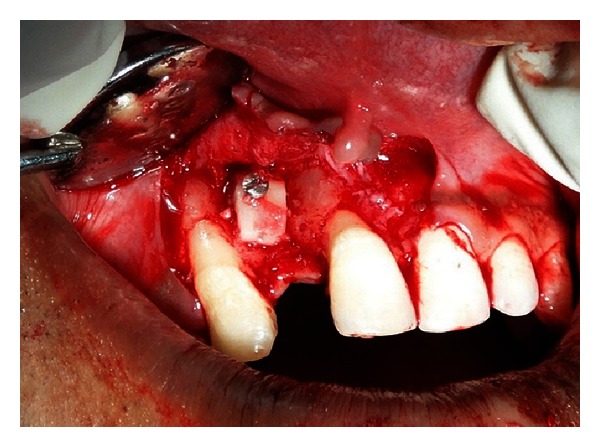
Block graft fixed at the recipient site.

**Figure 9:**
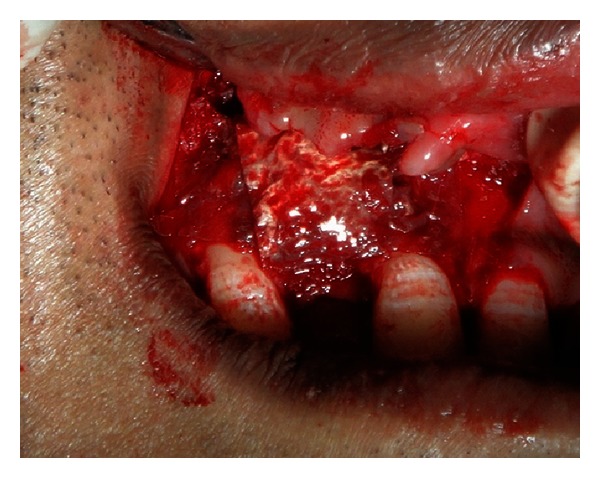
Chorion membrane placed over graft.

The recipient site was perforated with a fissure bur to induce bleeding and promote revascularization of the graft. The harvested block was adapted to the recipient site and held in position with a self-tapping screw (Figure 8). Edges of the graft were smoothened to avoid trauma to overlying flap. A chorion membrane (Tata Memorial Hospital Tissue Bank, Mumbai) was used to cover the area after DFDBA was placed on the periphery of the block (Figure 9). Interrupted 3-0 silk sutures were placed to close the site. Analgesics were prescribed, and postoperative instructions were given to patient.

**Figure 10:**
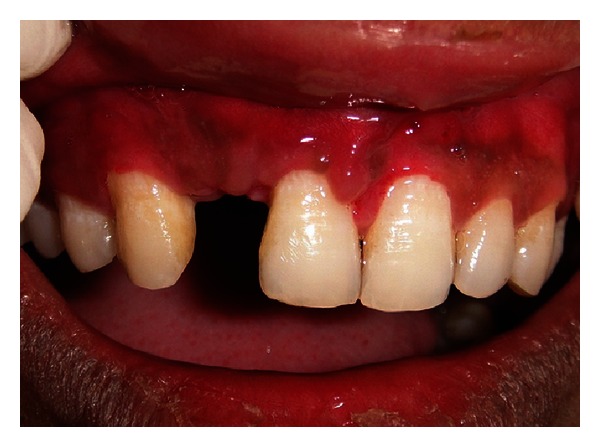
Edentulous area 3 weeks after graft placement.

Soft tissue healing at both sites was uneventful (Figure 10). Sutures were removed after 15 days.

5 months later, ridge mapping showed buccolingual width as 7.3 mm, 3 mm apical to the alveolar crest. A panoramic radiograph was obtained to evaluate the grafted area. A surgical reentry with implant placement in a single visit was planned.

#### 2.1.4. Surgical Reentry and Implant Placement

**Figure 11:**
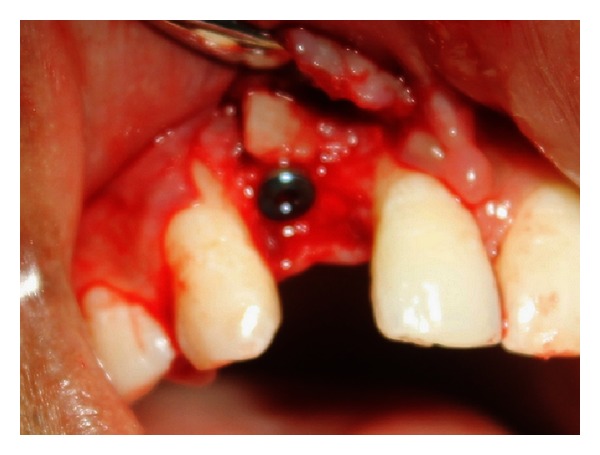
Implant placed in the augmented site.

A crestal incision was given after administering infraorbital nerve block (Lignocaine 2% with Adrenaline 1 : 80,000), followed by mucoperiosteal flap elevation to expose the grafted site. Buccolingual width of the ridge was found to be 6.9 mm. Fixation screw was removed, and osteotomy was done for implant placement. A 3.3 × 11.5 mm implant (Alpha-Bio Tec Ltd., Israel) was inserted (Figure 11). Primary stability was measured using Osstell device that showed Implant Stability Quotient (ISQ) value as 70. Interrupted sutures were given after placing cover screws and adaptation of flap. Postoperative period was uneventful. Sutures were removed on the 7th day.

#### 2.1.5. Implant Exposure and Prosthesis Placement

Second stage implant surgery was done 6 months later. After topical application of anaesthetic gel (Lignocaine 2%) the mucosa overlying implant was removed with Er, Cr:YSGG laser (Waterlase YSGG C 100, Biolase Technology, USA), followed by placement of healing cap for 15 days. Impressions were made for prosthesis fabrication, followed by metal ceramic crown placement, completing the prosthetic rehabilitation. 

### 2.2. Clinical Outcome

**Figure 12:**
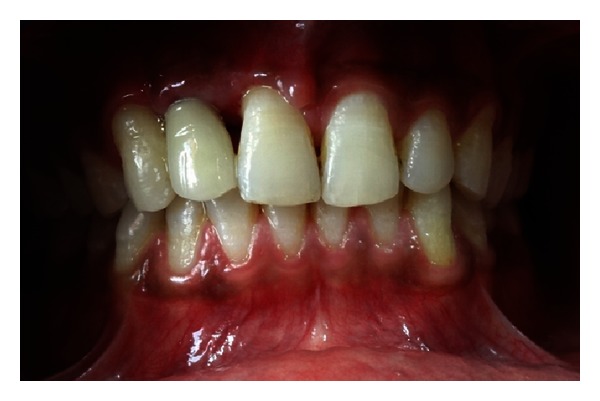
Restored implant 1 year after loading.

**Figure 13:**
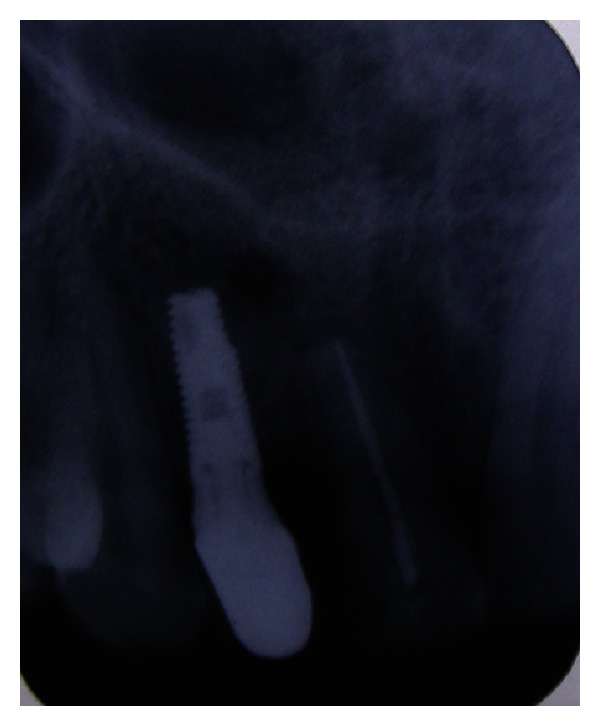
IOPA radiograph of the restored site 1 year after loading.

This case is being followed regularly for last 1 year after crown placement, and no complication has been reported till now (Figures 12 and 13). The prosthesis is functioning without any discomfort to the patient. IOPA radiograph taken 1 year after loading shows no evidence of bone loss around the implant.

## 3. Discussion

Adequate bone volume at the future implant site is a prerequisite for good aesthetic outcome and sound biomechanical support of the osseointegrated implant. However, alveolar deficiencies due to trauma and subsequent unpredictable patterns of bone resorption may prevent an ideal implant therapy for prosthodontic rehabilitation. Identification of the exact anatomy of the edentulous site is essential before planning an implant placement. As in the present case, it was difficult to predict the morphology of underlying bone, since the only information we got through clinical examination was a concavity on the labial aspect of edentulous area. Radiographic evaluation thus became necessary to get an idea of the size and shape of underlying defect. Two-dimensional radiographs, such as Intraoral Periapical (IOPA) or panoramic views, could not reveal the exact condition of labial cortical plate. To get a cross-sectional view of the site, a 3-dimensional technique was required. Conventional computed tomography (CT) fulfils this requirement very well, but the radiation dose is much higher than cone beam computed tomography (CBCT) which gives better view with 40 to 60 times less radiation [5]. A slight concavity on the labial aspect of the ridge found on clinical examination, turned out to be a gross defect on CBCT evaluation. 

Various modalities have been reported to successfully augment the alveolar ridge in such cases. These include inlay or onlay block grafts and guided bone regeneration.

As compared to onlay grafts, particulate grafts require additional materials to ensure space maintenance and graft containment such as barrier membranes, tenting screws, and/or graft binders, while onlay grafts are self-contained and provide an inherent ability to support the soft tissue [4]. Therefore, onlay autografts are the best grafting material for ridge augmentation procedure. Iliac crest graft was first to be used to increase mandibular alveolar bone height in 1951. More recently developed procedures harvested cortico-cancellous bone grafts from the mandibular symphysis and the buccal ramus areas [3, 6–8]. 

Because of high risk associated with harvesting iliac crest bone and life-threatening complications with calvarial bone, intraoral source has been suggested to be a safer and easier option when amount of bone is not a concern. Additionally, harvesting graft from extraoral sources requires general anaesthesia and hospital admission with a longer healing period which can be avoided when an intraoral site is chosen [3, 4]. Several studies have shown that intraoral autogenous grafts have a lower rate of resorption and better revascularization compared to iliac crest grafts [4]. 

In this case, mandibular symphysis was chosen to be the donor site, as it is easily accessible for harvesting block grafts, and we needed a small block of bone. It has advantages of considerably less discomfort, no visible scar formation, no need of general anaesthesia, and faster healing. When planning a block graft harvesting from mandibular symphysis, CBCT has another important role to play. Labiolingual width of the mandible and depth of the lingual concavity have to be accurately assessed, which is not possible with two-dimensional imaging modalities. We found CBCT an invaluable tool for treatment planning in this case.

The surgical procedure was easily done under local anaesthesia with a short recovery period and uneventful healing at the donor site as in majority of the reported cases [4, 7]. 

Piezoelectric unit was used for harvesting of block, as it is a minimally invasive technique with lesser risk of damage to surrounding mucosa, nerves, or vessels. Rapid cutting by piezoelectric unit prevents increase in temperature at surgical site, thus maintaining osteogenic potential of bone cells. It requires much less hand pressure than rotary instruments for its cutting action, providing more tactile sensitivity and accuracy to the operator. When used under local anaesthesia, it is more acceptable to patients compared to conventional surgical burs or saws [8, 9]. 

Using a particulate graft such as DFDBA helps bridging the minute gaps between cortical block and native bone, thus resulting in a more uniform and aesthetic alveolar ridge, in addition to providing osteoinductive effect [10, 11]. Particulate grafts also have the advantage of rapid vascularization; however, they must be protected by a membrane to reduce the risk of resorption.

As it has been suggested that block and particulate grafts show less resorption when covered with a barrier membrane through the healing period [12], a chorion membrane was placed in the present case. It is composed of immunoprivileged tissues and offers various advantages over alloplastic membranes. These membranes possess antibacterial and antimicrobial properties, reduce inflammation at the wound site, and provide a protein enriched matrix to facilitate cell migration [13].

Surgical reentry and implant placement was done 5 months after placement of block graft as Pikos suggested that block grafts can be reentered at 3-4 months in the mandible and at 4-5 months in the maxilla [14]. 

Primary stability of the implants placed in the grafted bone can be easily assessed at the time of placement using resonance frequency analysis (RFA). In the present case, ISQ value was found to be 70 which indicates good primary stability.

In this case, the implant has been free of any complications after one year of placement in accordance with several studies reporting high success rates of implants placed in onlay grafts. Clementini et al. [7] reported that success rate of implants placed in onlay graft regenerated ridges ranged from 72.8% to 97% after follow-up periods ranging from 6 months to 10 years, very similar to those obtained in case of implants placed in pristine bone, and suggested that onlay graft augmentation is a quite predictable technique to allow the placement of implants in severely atrophic areas. In a systematic review, Aghaloo and Moy [6] reported implant survival rate as 95.5% for guided bone regeneration, 90.4% for onlay/veneer grafting, 94.7% for distraction osteogenesis, and 83.8% for combinations of onlay, veneer, and interpositional inlay grafting.

## 4. Conclusion

Well-functioning implant after 1-year followup indicates that bone lost to trauma in maxillary anterior region can be successfully restored with an onlay block graft from mandibular symphysis, without much morbidity and complications. Cone beam computed tomography (CBCT) is invaluable tool in diagnosis and treatment planning of such cases.
